# Maternal smoking, nutritional factors at different life stage, and the risk of incident type 2 diabetes: a prospective study of the UK Biobank

**DOI:** 10.1186/s12916-024-03256-8

**Published:** 2024-02-02

**Authors:** Wenbo Jiang, Yiwei Tang, Ruiming Yang, Yujia Long, Changhao Sun, Tianshu Han, Wei Wei

**Affiliations:** 1 Key Laboratory of Precision Nutrition and Health, Ministry of Education, Department of Nutrition and Food Hygiene, the National Key Discipline, School of Public Health, Harbin Medical University, Harbin, P. R. China; 2 Department of Cardiology, the First Affiliated Hospital of Harbin Medical University, Harbin, China; 3 Department of Pharmacology, College of Pharmacy Key Laboratory of Cardiovascular Research, Ministry of Education, Harbin Medical University, Harbin, P. R. China

**Keywords:** Maternal smoking, Breastfeeding, Nutritional factors, Genetic susceptibility, Type 2 diabetes

## Abstract

**Background:**

This study aims to investigate potential interactions between maternal smoking around birth (MSAB) and type 2 diabetes (T2D) pathway-specific genetic risks in relation to the development of T2D in offspring. Additionally, it seeks to determine whether and how nutritional factors during different life stages may modify the association between MSAB and risk of T2D.

**Methods:**

This study included 460,234 participants aged 40 to 69 years, who were initially free of T2D from the UK Biobank. MSAB and breastfeeding were collected by questionnaire. The Alternative health eating index(AHEI) and dietary inflammation index(DII) were calculated. The polygenic risk scores(PRS) of T2D and pathway-specific were established, including β-cell function, proinsulin, obesity, lipodystrophy, liver function and glycated haemoglobin(HbA1c). Cox proportion hazards models were performed to evaluate the gene/diet-MSAB interaction on T2D. The relative excess risk due to additive interaction (RERI) were calculated.

**Results:**

During a median follow-up period of 12.7 years, we identified 27,342 cases of incident T2D. After adjustment for potential confounders, participants exposed to MSAB had an increased risk of T2D (HR=1.11, 95%CI:1.08-1.14), and this association remained significant among the participants with breastfeeding (HR= HR=1.10, 95%CI: 1.06-1.14). Moreover, among the participants in the highest quartile of AHEI or in the lowest quartile of DII, the association between MSAB and the increased risk of T2D become non-significant (HR=0.94, 95%CI: 0.79-1.13 for AHEI; HR=1.09, 95%CI:0.99-1.20 for DII). Additionally, the association between MSAB and risk of T2D became non-significant among the participants with lower genetic risk of lipodystrophy (HR=1.06, 95%CI:0.99-1.14), and exposed to MSAB with a higher genetic risk for β-cell dysfunction or lipodystrophy additively elevated the risk of T2D(RERI=0.18, 95%CI:0.06-0.30 for β-cell function; RERI=0.16, 95%CI:0.04-0.28 for lipodystrophy).

**Conclusions:**

This study indicates that maintaining a high dietary quality or lower dietary inflammation in diet may reduce the risk of T2D associated with MSAB, and the combination of higher genetic risk of β-cell dysfunction or lipodystrophy and MSAB significantly elevate the risk of T2D in offspring.

**Supplementary Information:**

The online version contains supplementary material available at 10.1186/s12916-024-03256-8.

## Background

Type 2 diabetes (T2D) has become a significant worldwide public health concern due to its rapidly increasing prevalence, elevated rates of mortality and disability, resulting in a substantial global burden on both healthcare and the economy [1]. Therefore, the identification of potential risk factors is a crucial task for the prevention and treatment of T2D. Some observational studies have documented a link between maternal smoking around birth (MSAB) and an increased risk of T2D [2, 3]. However, the specific disease pathways involved in this link remain largely unknown, and limited research has explored the possibility of behavioral interventions at various life stages to reduce the risk of T2D associated with MSAB.

Research has found that MSAB can disrupt pancreatic development and function through mitochondrial-mediated β cell apoptosis with higher levels of inflammation and oxidative stress in offspring [4–8]. Therefore, nutritional factors that can alleviate these detrimental conditions may be more important for subjects who experienced MSAB. However, few studies have examined the efficacy of such nutritional factors in preventing T2D among this specific group. Breastfeeding and overall dietary quality are important nutritional factors at early life and throughout adulthood. Previous studies have suggested that breastfeeding can enhance offspring insulin sensitivity and secretion, while reducing inflammation and oxidative stress levels [9, 10]. Moreover, a substantial body of evidence has linked overall dietary quality to insulin sensitivity, insulin secretion, inflammation, and oxidative stress [11–15]. Building upon this existing knowledge, our study postulates that early-life breastfeeding and a sustained commitment to a high-quality diet into adulthood may mitigate the risk of T2D among individuals exposed to MSAB.

Furthermore, the recent rapid advancements in gene-sequencing technologies have not only facilitated the identification of individuals at high risk for T2D but have also created significant opportunities for investigating potential interactions between risk factors and disease-specific genetic pathways [16]. A recent soft clustering analysis of T2D-associated loci has successfully categorized them into five distinct clusters, each representing a probable disease-causing pathway, including β-cell, proinsulin, obesity, lipodystrophy, and liver/lipid clusters [17]. Therefore, examining the potential interactions of MSAB with these pathway-specific genetic loci may add more important knowledge for the underlying the association between MSAB and T2D.

Therefore, this study aims to prospectively examine whether and how breastfeeding or overall dietary quality would influence the association between MSAB and risk of T2D. Additionally, this study also aims to evaluate the potential interactions of maternal smoking during pregnancy with these pathway-specific genetic loci of T2D.

## Methods

### Study population

The UK Biobank is a large study that includes over 500,000 participants who joined the study between 2006 and 2010, with an age range of 40-69 years. Detailed information about the study design and methods has been previously published [18]. During the recruitment phase, participants provided information through questionnaires on various socio-demographic factors, health status, medical history, family background, and lifestyle choices. Additionally, physical measurements were taken. All participants provided written consent to participate in the UK Biobank study, which was approved by the North West Multi-Centre Research Ethics Committee. For our analysis, we focused on participants with complete data for the variables of interest (*n*=472,301) and excluded those who had already been diagnosed with T2D at the time of enrollment (*n*=12,067). This resulted in a final sample size of 460,234 individuals after excluding participants with missing data related to the exposures of interest. Investigating the interaction between maternal smoking or breastfeeding and genetic risk score in relation to T2D was a key objective of our study.

### Assessment of exposure and outcome

Participants in the study provided information about maternal smoking around the time of their birth and whether they were breastfed as babies through a touchscreen questionnaire. The questionnaire included questions such as "Did your mother smoke regularly around the time when you were born?" (Data-Field 1787) and "Were you breastfed when you were a baby?" (Data-Field 1677). Participants could choose from response options including "Yes," "No," "Don't know," and "Prefer not to answer." Those who selected "Don't know" or "Prefer not to answer" were considered missing data and were excluded from the analysis.

The alternative healthy eating index was calculated based on 11 components: vegetables, fruits, whole grains, nuts/legumes, omega-3 fatty acids, polyunsaturated fatty acids, sugary beverages and fruit juices, red and processed meats, sodium, trans fatty acids and alcohol intake. Each component is scored on a scale of 0 to 10. A score of 10 indicates that the recommendations were fully met, whereas a score of 0 represents the least healthy dietary behavior. Intermediate intakes were scored proportionately between 0 and 10. The total score ranges from 0 to 110 [19].

DII scores were calculated based on the mean of the available Oxford WebQ dietary data using methodology described in a previous study [20]. Briefly, we included 18 foods and nutrients in the UK Biobank dataset to create the Dietary Inflammatory Index (DII): alcohol, carbohydrates, dietary fiber, folate, saturated fat, polyunsaturated fat, protein, total fat, vitamin B12, vitamin B6, iron, magnesium, vitamin C, vitamin E, tea, garlic, onions, and total energy. The scores specific to these 18 food parameters were summed to obtain the overall DII score.

To define prevalent and incident cases of T2D in the UKB cohort, we followed the algorithms developed by previous study. The diagnosis of T2D was based on self-reported medical history and medication information provided by the participants. The International Classification of Diseases, 10th edition (ICD-10) code E11 was used to define T2D cases. The prevalent T2D cases were identified if the participants reported a history of T2D, reported taking medication for T2D at the time of enrolment (such as insulin, sulphonylureas, glitazones, meglitinides, or acarbose), or if their HbA1c level was >48 mmol/mol (6.5%). For additional details, please refer to https://biobank.ctsu.ox.ac.uk/showcase/label.cgi?id=2000.

### Details of covariates

The baseline touch-screen questionnaire was used to assess potential confounders, including age(years), sex(male/female), race(non-white/white), smoking status(never, previous and current), drinking status(never, previous and current). Physical activity (metabolic equivalents minutes per week were calculated according to the International Physical Activity Questionnaire short form: 1 min walking=3.3 METS, 1min moderate physical activity=4 METS and 1 min vigorous physical activity=8 METS), employment status (employed/retired/others), BMI (<25, 25-29.9, or 30 kg/m^2^), birth weight (deciles), hypertension drug use (yes/no), cholesterol-lowering drug use (yes/no), and family history of diabetes (yes/no). Townsend Index of Deprivation is a measure of socioeconomic status (SES), derived using census data and participants' postcodes, where higher scores represent higher levels of deprivation.

### Polygenic risk score(PRS) for T2D

The genotyping process, quality control measures, and imputation procedures for the UKB have been previously described in detail [21]. In our study, we utilized a set of 424 single nucleotide polymorphisms (SNPs) that passed quality control to derive the PRS for T2D [22]. For each SNP, the individual's score was determined by the number of risk alleles they carried (0, 1 or 2). The SNP scores were then weighted based on the effect size of each SNP on T2D, as reported in a genome-wide association study (GWAS) conducted by Scott et al. in individuals of European descent [22]. Finally, the weighted sum of the risk alleles was calculated using the following formula: PRS=(β_1_×SNP_1_+β_2_×SNP_2_ +...+β_102_×SNP_102_) × (424/sum of the β coefficients), where βi represents the effect value of the ith SNP obtained from the aforementioned GWAS study results. To investigate potential interactions between established T2D genetic loci and maternal smoking, we constructed five pathway-specific genetic risk scores based on a recent soft clustering analysis of T2D-associated loci. This clustering analysis identified five distinct clusters that likely represent disease-causing pathways: β-cell, proinsulin, obesity, lipodystrophy, and liver/lipid clusters, as described in previous studies [16, 17]. Moreover, we also included the genetic risk score of HbA1c based on the initial release of PRS in the UK biobank [23].

### Statistical analysis

All analyses were conducted using R 4.2.1. and *P*<0.05 were statistically significant. Baseline characteristic for continuous variables were expressed as means (standard deviation, SD) and categorical variables were expressed as numbers (percentages, %), which were compared by general linear model and logistic regression model with adjustment for age and sex. The cox proportional hazards (CPH) models were used to evaluate the association interaction of the MSAB with AHEI, DII and PRS on risk of T2D. Survival time was from participation in the UKB assessment center to diagnosis of T2D or up to February 1, 2022. Our CPH models included several covariates: age, sex, race, smoking status, alcohol consumption, employment status, BMI, physical activity level, Townsend deprivation index, birth weight, hypertension drug use, cholesterol-lowering drug use, and family history of diabetes. In cases where covariate information was missing, we imputed the mean for continuous variables or used the missing indicator method for categorical variables. To explore potential modification effects of AHEI, DII and PRS on the association between MSAB and risk of T2D, we categorize the AHEI, DII and each PRS by quartiles from bottom to top, and respectively examine the association between MSAB and risk of T2D in the different quartiles. We also additionally added the interaction terms into the CPH models to calculate the *P*-values of the interaction terms. To examine the additive association of AHEI, DII and PRS between MSAB on risk of T2D, the cross-groups terms based on the MSAB and quartiles of AHEI, DII and each PRS were created, which were added in the CPH model. Participants without the MSAB and in the lowest quartile of AHEI, DII and each PRS were set as the reference group. Meanwhile, the relative excess risk due to interaction (RERI) and the attributable proportion due to interaction(AP) were calculated to evaluate the statistical significance of the additive association. An absence of additive interaction was indicated when the confidence intervals of RERI and AP included zero.

### Sensitivity analysis

Three sensitivity analyses to ensure the robustness of our findings. Firstly, to minimize the impact of reverse causation, we excluded participants with less than 2 years of follow-up. Secondly, to mitigate confounding effects from personal smoking, we restricted our analysis to nonsmoking participants. Thirdly, to exclude the influence of selection bias on our results, we performed a propensity score matching (PSM) for the participants without information of maternal smoking and breastfeeding. The PSM method was matched by 12 covariables for baseline characteristics. The nearest neighbor matching within a specified caliper distance was used as the criteria for selecting participants with information of maternal smoking and breastfeeding with calipers of width equal to 0.2.

## Results

### Baseline characteristics of participants

Table 1 provides an overview of the initial characteristics of the study cohort, categorized according to the presence or absence of T2D. Among these participants, a total of 5.9% of the study population have T2D during the follow-up. Individuals with T2D tended to be older, predominantly male, and of white ethnicity. Additionally, this group displayed a heightened prevalence of obesity, reduced engagement in physical activity, elevated Townsend Deprivation Index scores, a higher frequency of maternal smoking, and diminished rates of breastfeeding.

**Table 1:** Baseline characteristics of participants by T2D in the UK Biobank cohort

	**Incident T2D**		***P* value**
	**No**	**Yes**	
Participants (%)	432,892 (94.1)	27,342 (5.9)	
Age (years), mean (SD)	56.23 (8.10)	59.28 (7.26)	<0.001
Gender (%)			<0.001
Female	242,579 (56.0)	11,527 (42.2)	
Male	190,313 (44.0)	15,815 (57.8)	
Race (%)			<0.001
Non-white ethnicity	18,293 (4.2)	2,715 (9.9)	
White ethnicity	414,599 (95.8)	24,627 (90.1)	
Smoking (%)			<0.001
Never	241,736 (55.8)	12,257 (44.8)	
Previous	147,630 (34.1)	11,362 (41.6)	
Current	43,526 (10.1)	3,723 (13.6)	
Drinking (%)			<0.001
Never	16,415 (3.8)	1,978 (7.2)	
Previous	13,775 (3.2)	1,637 (6.0)	
Current	402,702 (93.0)	23,727 (86.8)	
Employment (%)			<0.001
In paid employment or self-employed	258,051 (59.6)	12,113 (44.3)	
Retired	139,782 (32.3)	11,705 (42.8)	
Others	35,059 (8.1)	3,524 (12.9)	
BMI (%), kg/m^2^			<0.001
<25	153,916 (35.6)	2,611 (9.5)	
25-29.9	186,911 (43.2)	9,724 (35.6)	
≥30	92,065 (21.3)	15,007 (54.9)	
MET-min/week ≥600 (%)	278,904 (64.4)	14,673 (53.7)	<0.001
≥600			
Townsend deprivation index, mean (SD)	-1.46 (3.00)	-0.58 (3.37)	<0.001
Birth weight, mean (SD), kg	3.33 (0.66)	3.24 (0.76)	<0.001
Hypertension drug use (%)	23,609 (5.5)	2,058 (7.5)	<0.001
Cholesterol-lowering drug use (%)	24,123 (5.6)	4,592 (16.8)	<0.001
Family history of diabetes (%)	74,215 (17.1)	8,865 (32.4)	<0.001
Maternal smoking around birth (%)	109,657 (25.3)	7,343 (26.9)	<0.001
Breastfed as a baby (%)	241,387 (55.8)	14,925 (54.6)	<0.001

Continuous variables are presented as Mean(SD). Categorical variables are presented as numbers (%, percentage)

*T2D* Type 2 diabetes, *BMI* Body mass index, *SD* Standard deviation

### Modification and joint effects of nutritional factors on the association between MSAB and risk of T2D

**Fig. 1:**
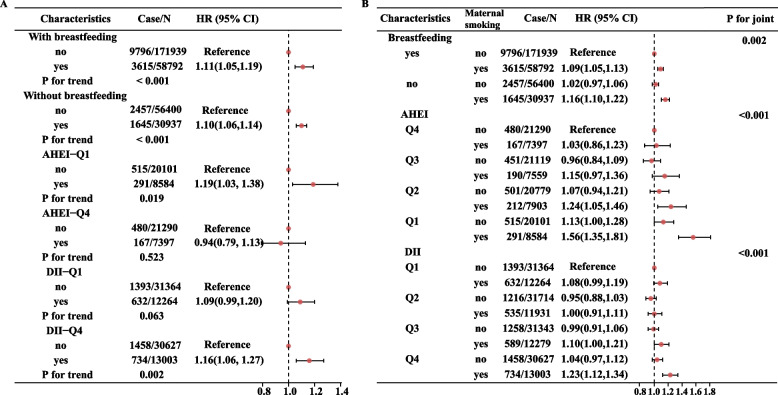
The association between MSAB and T2D stratified by various levels of breastfeeding, AHEI and DII

During a median follow-up of 12.7 years, we observed 27,342 incident cases of T2D. After adjustment for potential confounders, as indicated by HRs and 95%CI, compared with other participants, the participants exposed to MSAB had a higher risk of developing T2D (HR=1.11, 95%CI:1.08-1.14); while participants with breastfeeding had lower risk of T2D (HR=0.95, 95%CI:0.92-0.99) (Additional file 1: Table S1). Meanwhile, compared with participants in the lowest quartile of AHEI, participants in the highest quartile had lower risk of T2D (HR=0.84, 95%CI:0.76-0.94), and participants in the highest quartile of DII had higher risk of T2D (HR=1.08, 95%CI:1.01-1.14) (Additional file 1: Table S1). The modification effects indicated by HRs and 95%CI were presented in Fig. 1A. Among the participants with breastfeeding, exposed to MSAB was still associated with an increased risk of T2D (HR=1.10, 95%CI:1.06-1.14). Meanwhile, among the participants in the highest quartile of AHEI, the association between MSAB and the increased risk of T2D become non-significant (HR=0.94, 95%CI: 0.79-1.13); while among the participants in the highest quartile of DII, such association was still significant (HR=1.16, 95%CI:1.06-1.27).

The joint effects indicated by HRs and 95%CI were presented in Fig. 1B. Although compared with participants who received breastfeeding and did not experience MSAB, the participants exposed to MSAB without breastfeeding had a greater risk of T2D (HR=1.16, 95%CI:1.10-1.22), the additive association indicated by RERI and AP was non-significant (RERI=-0.04, 95%CI: -0.19-0.10; AP=-0.04, 95%CI: -0.19-0.10) (Additional file 1: Table S2). Similarly, compared to participants in the highest quartile of AHEI without MSAB, participants in the lowest quartile of AHEI with MSAB had an increased risk of T2D (HR=1.56, 95%CI: 1.35-1.81), and compared to participants in the lowest quartile of DII without MSAB, participants in the highest quartile of DII with MSAB had an increased risk of T2D (HR=1.23, 95%CI: 1.12-1.34); however, only the additive association between MSAB and AHEI was non-significant (RERI=-0.29, 95%CI:-0.55 to -0.03; AP=-0.28, 95%CI:-0.56 to -0.01) (Additional file 1: Table S2).

### Modification and joint effects of genetic susceptibility on the association between MSAB and risk of T2D

**Fig. 2:**
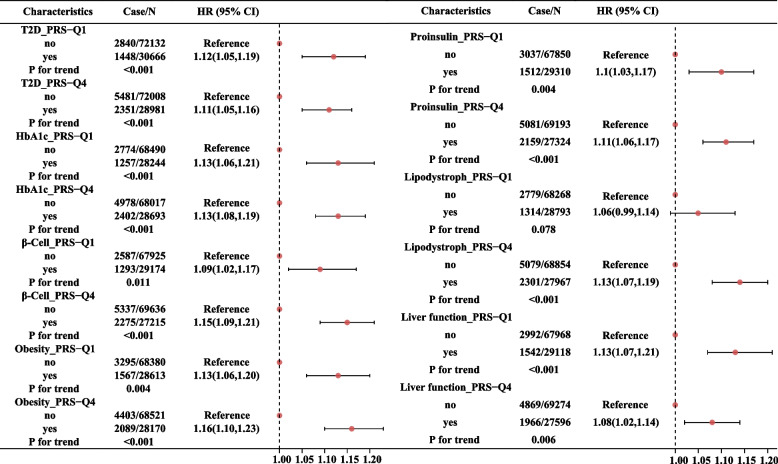
The association between MSAB and T2D stratified by different genetic risk scores

A higher PRS of T2D and disease-causing genetic pathway in terms of HbA1c deposition, β-cell function, proinsulin synthesis, obesity, lipodystrophy, and liver function were all significantly associated with an increased risk of T2D (Additional file 1: Table S3). The modification effects of these PRS indicated by HRs and 95%CI were presented in Fig. 2. The association between MSAB and risk of T2D become non-significant only among the participants in the lowest quartile of lipodystrophy (HR=1.06, 95%CI:0.99-1.14), and such association was still significant among participants in the lowest quartile of other PRS (HR=1.12, 95%CI:1.05-1.19 for T2D-PRS; HR=1.13, 95%CI:1.06-1.21 for HbA1c deposition; HR=1.09, 95%CI:1.02-1.17 for β-cell function; HR=1.13, 95%CI: 1.06-1.20 for obesity; HR=1.10, 95%CI:1.03-1.17 for proinsulin synthesis; HR=1.13, 95%CI:1.07-1.21 for liver function).

**Fig. 3:**
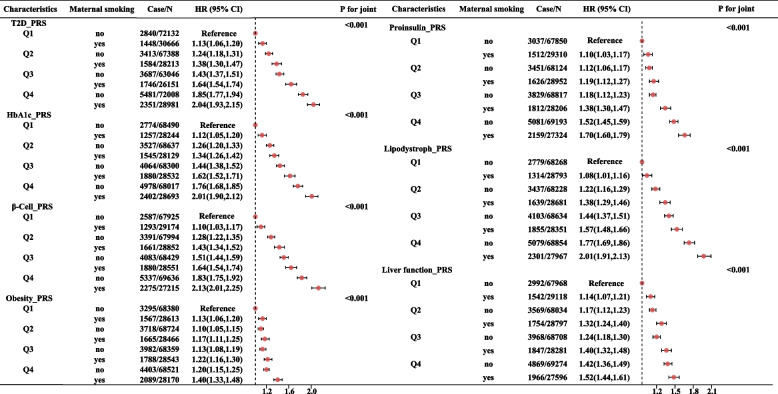
Joint analysis of genetic risk scores and MSAB in relation to T2D

The joint effects of these PRS and MSAB indicated by HRs and 95%CI were presented in Fig. 3. Compared to participants without MSAB and in the lowest quartile of PRS, participants in the highest quartile of PRS with MSAB had an increased risk of T2D (HR=2.04, 95%CI:1.93-2.15 for T2D-PRS; HR=2.01, 95%CI:1.90-2.12 for HbA1c deposition; HR=2.13, 95%CI: 2.01-2.25 for β-cell function; HR=1.40, 95%CI: 1.33-1.48 for obesity; HR=1.70, 95%CI:1.60-1.79 for proinsulin synthesis; HR=2.01, 95%CI: 1.91-2.13 for lipodystrophy; HR=1.52, 95%CI:1.44-1.61 for liver function); however, as indicated by RERI and AP (Additional file 1: Table S2), only the additive association of β-cell function (RERI=0.18, 95%CI:0.06-0.30; AP=0.08, 95%CI:0.03-0.14) and lipodystrophy (RERI=0.16, 95%CI:0.04-0.28; AP=0.08, 95%CI:0.02-0.14) with MSAB was significant.

### Sensitivity analysis

Three sensitivity analyses were performed. The first sensitivity analysis showed that after excluding the participants who were diagnosed by T2D in the two years follow-up, and we still observed these above results, suggesting the acute occurrence of T2D did not influence the results (Additional file 2: Figs. S1-3). Secondly, we found that the above results did not change significantly when we restricted the analysis to nonsmokers (Additional file 2: Figs. S4-6), suggesting that smoking behavior in the offspring did not influence our results. Lastly, the participants with missing information of MSAB and breastfeeding were more likely to be old, retired women, smokers and drinkers (Additional file 1: Table S4). After the PSM, no-significant differences were found between groups with and without information of maternal smoking and breastfeeding (Additional file 1: Table S5). We still observed that maintaining a high dietary quality or lower dietary inflammation foods intake may reduce the risk of T2D associated with MSAB and the combination of maternal smoking around birth and higher genetic risk of β-cell function or lipodystrophy additively elevated the risk of developing T2D among the sample without any differences of baseline characteristics with the excluding sample in the main analysis (Additional file 2: Figs. S7-9).

## Discussion

In this prospective cohort study, we found that participants exposed to MSAB had a higher risk of developing T2D. Notably, early-life breastfeeding did not alter this association. However, individuals who consistently maintained a high AHEI score or a low DII score throughout adulthood experienced a reduced risk of T2D, even if they had been exposed to MSAB. Further, this association remained consistent among individuals with lower genetic risk for T2D, β-cell function, proinsulin synthesis, obesity, liver function and HbA1c deposition, but it attuned among the participants with lower genetic risk of lipodystrophy. Additionally, a higher PRS of β-cell function and lipodystrophy with the MSAB additively elevated risk of T2D.

Smoking during pregnancy in women is an important public health issue. It is estimated that the prevalence of maternal smoking during pregnancy is 30.6% in the European Region [24]. Consistent with these data, a total of 25.4% of participants reported that their mothers smoked around their born in the data of UK biobank. Accumulating studies have documented detrimental health impacts of MSAB on offspring, such obesity, hypertension and cancer [25–28], and a recent study also augment this link to higher incidence of T2D in offspring [3]. These studies emphasized the importance of smoking cessation during pregnancy. However, nearly half of female smokers do not quit smoking during pregnancy, possible because of nicotine addiction, living stress and etc. [29], which may bring serious health burden in the offspring. Therefore, identifying modifiable factors that can mitigate these deleterious effects is an important task for public health research.

In this study, we focused on the nutritional factors at different life stages for modifying the association between MSAB and risk of T2D because of its feasible and cost-effective benefits [30]. A few studies have emphasized the importance of breastfeeding during the early life on metabolism of energy and glucose because of abundant bioactive components in breast milk [31–33], and it shares common biological pathways with MSAB. However, we found that breastfeeding did not mitigate the link between MSAB and the increased risk of T2D, suggesting that mothers who smoked during pregnancy may also influence the function of breast milk, impairing its beneficial effects. This observation may be attributed to the additional exposure to tobacco-derived compounds present in breast milk. Smoking constitutes a habit characterized by addiction, often proving challenging to cease abruptly [34]. Mothers who smoke during pregnancy or encounter second-hand smoke exposure frequently persist in such behaviors postpartum [35]. Consequently, nicotine and other constituents found in tobacco can be conveyed to the fetus via breast milk, resulting in indirect fetal exposure [36]. Further investigations are warranted to delve into the extent to which individuals exposed to maternal smoking might derive benefits from breastfeeding in comparison to those without maternal smoking exposure.

Further, we observed that under the condition of MSAB, the participants who commitment to a high-quality diet or low inflammation foods in their daily diet during adulthood had lower risk of developing T2D, suggesting that the nutritional behavior during adulthood aided in alleviating the deleterious effects of maternal smoking during pregnancy on glucose homeostasis. These observational could be partially supported by previous studies. It has been documented that substances contained in tobacco smoke, such as nicotine, carbon monoxide, and polycyclic aromatic hydrocarbons, could cross the placenta, enter the fetal circulation [5, 37], and further interfere with their development and induce permanent alterations in metabolism. Additionally, other toxic constituents in cigarette smoke including aromatic compounds, heavy metals, and carbon monoxide could also interfere with glycometabolism by promoting inflammation and oxidative stress [9, 10]. Most of these biological mechanisms can be inhibited by improving overall dietary quality or low inflammation foods intake [11–15]. Collectively, these findings emphasized the importance for elevating the overall dietary quality among participants who experienced MSAB.

Moreover, the detail mechanism underlying the link between MSAB and the disordered of glucose homeostasis is still largely unknown, probably because it is difficult to establish an experimental animal model that mimic this condition. Therefore, we examined the modification and additive effects of causal disease-specific genetic risk score on the association between MSAB and risk of T2D. We found that only among the participants with lower genetic risk of lipodystrophy, the association between MSAB and the increased risk of developing T2D attuned, and other genetic risk in terms of β-cell function, proinsulin synthesis, obesity, liver function, and HbA1c deposition did not have such modification effects. Meanwhile, the combination of higher genetic risk for lipodystrophy and β-cell dysfunction with MSAB additively increased the risk of developing T2D. These observations suggest that genetic loci related to lipodystrophy and β-cell function may involve in regulating this association. Previous study has documented that the lipodystrophy-specific type of T2D is more likely to be characteristics as higher levels of triglycerides and insulin resistance, and β-cell dysfunction-specific type of T2D is more likely to be characteristics as impairment of proinsulin synthesis and insulin secretion, compared with other causal-specific pathway of genetic risk [17]. This finding prompted that decreasing hyperglycemia, insulin resistance and improving insulin secretion may be potential intervention targets for alleviating this deleterious effect. A previous study has found that parental smoking during pregnancy is associated with an increased risk of gestational diabetes in the daughter with higher levels of insulin resistance [38], and MSAB can disrupt pancreatic development and function through mitochondrial-mediated beta cell apoptosis [6], which may further support the findings in this study.

The major strengths of our knowledge include large sample size and prospective design. Its novelty lies in identifying potential modifiable factors that can mitigate the deleterious effects of MSAB on the development of T2D. Besides, we also employed a comprehensive analysis of T2D-related causal disease-specific PRS to explore the potential biological mechanisms underlying this link. However, it is important to acknowledge the potential limitations of this study. Firstly, due to its observational nature, the association between MSAB or breastfeeding and the risk of incident T2D cannot be interpreted as a causal relationship. Randomized clinical trials are necessary to establish causality and validate our findings. Secondly, some incident T2D cases relied on secondary diagnoses, which might result in a delay in identifying the actual onset time of T2D. Thirdly, the data collected on MSAB and breastfeeding was limited to binary information, indicating whether or not participants were exposed to MSAB or breastfeeding. Detailed information such as the amount and duration of smoking or breastfeeding was not available. Further studies should consider capturing these factors comprehensively to better understand their impact on the results. Fourthly, although we adjusted for major confounding factors, the possibility of residual confounding cannot be completely ruled out. Lastly, it should be noted that the study was conducted based on the UK Biobank dataset, which predominantly consists of participants of European descent. This might limit the generalizability of the results to other populations.

## Conclusion

In summary, our study emphasized the importance for maintain a high dietary quality or lower dietary inflammation foods intake among the individuals who experience maternal smoking during pregnancy. Moreover, the combination of higher genetic risk of β-cell dysfunction or lipodystrophy and MSAB significantly elevate the risk of T2D in offspring.

### Supplementary Information

**Additional file 1:** **Table S1.** The association between maternal smoking, breastfeeding, AHEI, DII and T2D. **Table S2.** Analysis of the additive interaction between maternal smoking and nutrition factors and T2D. **Table S3.** The association between genetic risk scores and T2D. **Table S4.** The differences for the baseline characteristics between participants with and without information of maternal smoking around birth and breastfeeding. **Table S5.** The differences for the baseline characteristics between participants with and without information of maternal smoking around birth and breastfeeding after PSM matching.

**Additional file 2:** **Fig. S1.** The association between MSAB and T2D stratified by various levels of breastfeeding, AHEI and DII, excluding individuals with follow-up periods less than two years. **Fig. S2.** The association between MSAB and T2D stratified by different genetic risk scores, excluding individuals with follow-up periods less than two years. **Fig. S3.** Joint analysis of genetic risk scores and MSAB in relation to T2D, excluding individuals with follow-up periods less than two years. **Fig. S4.** The association between MSAB and T2D stratified by various levels of breastfeeding, AHEI and DII in a non-smoking population. **Fig. S5.** The association between MSAB and T2D stratified by different genetic risk scores in a non-smoking population. **Fig. S6.** Joint analysis of genetic risk scores and MSAB in relation to T2D in a non-smoking population. **Fig. S7.** The association between MSAB and T2D stratified by various levels of breastfeeding, AHEI and DII in the selected population after propensity score matching. **Fig. S8.** The association between MSAB and T2D stratified by different genetic risk scores in the selected population after propensity score matching. **Fig. S9.** Joint analysis of genetic risk scores and MSAB in relation to T2D in the selected population after propensity score matching.

## Data Availability

The dataset used for this study will be uploaded to UK Biobank repository as per data user agreement with the UK Biobank. The dataset will be freely accessible via the repository subject to regulatory user approval from UK Biobank.

## Abbreviations

AHEI: Alternative health eating index
AP: Attributable proportion due to interaction
BMI: Body mass index
CPH: Cox proportional hazards
DII: Dietary inflammation index
GWAS: Genome-wide association study
HbA1c: Glycated haemoglobin
MSAB: Maternal smoking around birth
PRS: Polygenic risk scores
PSM: Propensity score matching
RERI: Relative excess risk due to interaction
SD: Standard deviation
SNPs: Single nucleotide polymorphisms
T2D: Type 2 diabetes

## References

[1] Global burden of 369 diseases and injuries in 204 countries and territories, 1990-2019: a systematic analysis for the Global Burden of Disease Study 2019. Lancet. 2020. 396(10258): 1204-1222. 10.1016/S0140-6736(20)30925-9 PMC7567026 33069326

[2] Jaddoe VW, de Jonge LL, van Dam RM, Willett WC, Harris H, Stampfer MJ (2014). Fetal exposure to parental smoking and the risk of type 2 diabetes in adult women. Diabetes Care.

[3] Ye Z, Li J, Gu P, Zhang Y, Xie Y, Yang S (2023). Early-life tobacco smoke exposure, genetic susceptibility and the risk of type 2 diabetes in adulthood: a large prospective cohort study. Sci Total Environ.

[4] Law CM, Barker DJ, Osmond C, Fall CH, Simmonds SJ (1992). Early growth and abdominal fatness in adult life. J Epidemiol Community Health.

[5] Bruin JE, Gerstein HC, Holloway AC (2010). Long-term consequences of fetal and neonatal nicotine exposure: a critical review. Toxicol Sci.

[6] Durlach V, Vergès B, Al-Salameh A, Bahougne T, Benzerouk F, Berlin I (2022). Smoking and diabetes interplay: a comprehensive review and joint statement. Diabetes Metab.

[7] Ye Z, Liang R, Wang B, Yu L, Liu W, Wang X (2022). Cross-sectional and longitudinal associations of urinary zinc with glucose-insulin homeostasis traits and type 2 diabetes: Exploring the potential roles of systemic inflammation and oxidative damage in Chinese urban adults. Environ Pollut.

[8] Zhang H, Han Y, Qiu X, Wang Y, Li W, Liu J (2020). Association of internal exposure to polycyclic aromatic hydrocarbons with inflammation and oxidative stress in prediabetic and healthy individuals. Chemosphere.

[9] Brink LR, Lönnerdal B (2020). Milk fat globule membrane: the role of its various components in infant health and development. J Nutr Biochem.

[10] George AD, Gay M, Selvalatchmanan J, Torta F, Bendt AK, Wenk MR (2021). Healthy Breastfeeding Infants Consume Different Quantities of Milk Fat Globule Membrane Lipids. Nutrients.

[11] Wang Z, Adair LS, Cai J, Gordon-Larsen P, Siega-Riz AM, Zhang B (2017). Diet quality is linked to insulin resistance among adults in China. J Nutr.

[12] Esfandiar Z, Hosseini-Esfahani F, Mirmiran P, Azizi F (2022). Diet quality indices and the risk of type 2 diabetes in the Tehran Lipid and Glucose Study. BMJ Open Diabetes Res Care.

[13] Wang YB, Page AJ, Gill TK, Melaku YA (2023). The association between diet quality, plant-based diets, systemic inflammation, and mortality risk: findings from NHANES. Eur J Nutr.

[14] Millar SR, Navarro P, Harrington JM, Perry IJ, Phillips CM (2021). Dietary quality determined by the healthy eating index-2015 and biomarkers of chronic low-grade inflammation: a cross-sectional analysis in middle-to-older aged adults. Nutrients.

[15] Aleksandrova K, Koelman L, Rodrigues CE (2021). Dietary patterns and biomarkers of oxidative stress and inflammation: a systematic review of observational and intervention studies. Redox Biol.

[16] Zhuang P, Liu X, Li Y, Li H, Zhang L, Wan X (2022). Circulating fatty acids and genetic predisposition to type 2 diabetes: gene-nutrient interaction analysis. Diabetes Care.

[17] Udler MS, Kim J, von Grotthuss M, Bonàs-Guarch S, Cole JB, Chiou J (2018). Type 2 diabetes genetic loci informed by multi-trait associations point to disease mechanisms and subtypes: a soft clustering analysis. PLoS Med.

[18] Conroy M, Sellors J, Effingham M, Littlejohns TJ, Boultwood C, Gillions L (2019). The advantages of UK Biobank's open-access strategy for health research. J Intern Med.

[19] Cornelis MC, Agarwal P, Holland TM, van Dam RM (2022). MIND dietary pattern and its association with cognition and incident Dementia in the UK biobank. Nutrients.

[20] Petermann-Rocha F, Wirth MD, Boonpor J, Parra-Soto S, Zhou Z, Mathers JC (2023). Associations between an inflammatory diet index and severe non-alcoholic fatty liver disease: a prospective study of 171,544 UK Biobank participants. BMC Med.

[21] Bycroft C, Freeman C, Petkova D, Band G, Elliott LT, Sharp K (2018). The UK Biobank resource with deep phenotyping and genomic data. Nature.

[22] Vujkovic M, Keaton JM, Lynch JA, Miller DR, Zhou J, Tcheandjieu C (2020). Discovery of 318 new risk loci for type 2 diabetes and related vascular outcomes among 1.4 million participants in a multi-ancestry meta-analysis. Nat Genet.

[23] DJ Thompson DW, S Selzam IP, Moore R. UK Biobank release and systematic evaluation of optimised polygenic risk scores for 53 diseases and quantitative traits. MedRxiv. 2022:2022-2026.

[24] Lange S, Probst C, Rehm J, Popova S. National, regional, and global prevalence of smoking during pregnancy in the general population: a systematic review and meta-analysis. Lancet Glob Health. 2018;6(7):e769-769e776. 10.1016/S2214-109X(18)30223-7. 10.1016/S2214-109X(18)30223-7 29859815

[25] Albers L, Sobotzki C, Kuß O, Ajslev T, Batista RF, Bettiol H (2018). Maternal smoking during pregnancy and offspring overweight: is there a dose-response relationship? An individual patient data meta-analysis. Int J Obes (Lond).

[26] Liang J, Fu Z, Liu Q, Shen Y, Zhang X, Weng Z (2022). Interactions among maternal smoking, breastfeeding, and offspring genetic factors on the risk of adult-onset hypertension. BMC Med.

[27] Rumrich IK, Viluksela M, Vähäkangas K, Gissler M, Surcel HM, Hänninen O (2016). Maternal smoking and the risk of cancer in early life - a meta-analysis. PLoS One.

[28] He H, He MM, Wang H, Qiu W, Liu L, Long L (2023). In utero and childhood/adolescence exposure to tobacco smoke, genetic risk, and lung cancer incidence and mortality in adulthood. Am J Respir Crit Care Med.

[29] Scherman A, Tolosa JE, McEvoy C (2018). Smoking cessation in pregnancy: a continuing challenge in the United States. Ther Adv Drug Saf.

[30] Downer S, Berkowitz SA, Harlan TS, Olstad DL, Mozaffarian D (2020). Food is medicine: actions to integrate food and nutrition into healthcare. BMJ.

[31] Ravelli AC, van der Meulen JH, Osmond C, Barker DJ, Bleker OP (2000). Infant feeding and adult glucose tolerance, lipid profile, blood pressure, and obesity. Arch Dis Child.

[32] Rich-Edwards JW, Stampfer MJ, Manson JE, Rosner B, Hu FB, Michels KB (2004). Breastfeeding during infancy and the risk of cardiovascular disease in adulthood. Epidemiology.

[33] Carrillo-Lozano E, Sebastián-Valles F, Knott-Torcal C (2020). Circulating microRNAs in breast milk and their potential impact on the infant. Nutrients.

[34] Lee H, Jeon Y, Yoo C, Seon H, Park J, Hwang M (2023). Persistent impacts of smoking on resting-state EEG in male chronic smokers and past-smokers with 20 years of abstinence. Sci Rep.

[35] Heinig MJ, Nommsen LA, Peerson JM, Lonnerdal B, Dewey KG (1993). Energy and protein intakes of breast-fed and formula-fed infants during the first year of life and their association with growth velocity: the DARLING Study. Am J Clin Nutr.

[36] Pettitt DJ, Forman MR, Hanson RL, Knowler WC, Bennett PH (1997). Breastfeeding and incidence of non-insulin-dependent diabetes mellitus in Pima Indians. Lancet.

[37] Lisboa PC, de Oliveira E, de Moura EG (2012). Obesity and endocrine dysfunction programmed by maternal smoking in pregnancy and lactation. Front Physiol.

[38] Bao W, Michels KB, Tobias DK, Li S, Chavarro JE, Gaskins AJ (2016). Parental smoking during pregnancy and the risk of gestational diabetes in the daughter. Int J Epidemiol.

