# Supplementary material for: Maternal smoking, nutritional factors at different life stage, and the risk of incident type 2 diabetes: a prospective study of the UK Biobank
Source: BMC Med. 2024 Feb 2;22:50. doi: 10.1186/s12916-024-03256-8 (PMC10835913; doi:10.1186/s12916-024-03256-8)
Supplement: Supplementary file 1 — Additional file 1: Table S1. The association between maternal smoking, breastfeeding, AHEI, DII and T2D. Table S2. Analysis of the additive interaction between maternal smoking and nutrition factors and T2D. Table S3. The association between genetic risk scores and T2D. Table S4. The differences for the baseline characteristics between participants with and without information of maternal smoking around birth and breastfeeding. Table S5. The differences for the baseline characteristics between participants with and without information of maternal smoking around birth and breastfeeding after PSM matching. [file 12916_2024_3256_MOESM1_ESM.docx]

S-Table 1. The association between maternal smoking, breastfeeding, AHEI, DII and T2D

| Characters | Model | no/Q1 | yes/Q2 | Q3 | Q4 |
| --- | --- | --- | --- | --- | --- |
| Maternal smoking |  |  |  |  |  |
|  | Model 1 | ref | 1.14 (1.11,1.17) |  |  |
|  | Model 2 | ref | 1.11 (1.08,1.14) |  |  |
| Breastfeeding |  |  |  |  |  |
|  | Model 1 | ref | 1.01 (0.97,1.04) |  |  |
|  | Model 2 | ref | 0.95 (0.92,0.99) |  |  |
| AHEI |  |  |  |  |  |
|  | Model 1 | ref | 0.86(0.78,0.95) | 0.76(0.69,0.85) | 0.77(0.69,0.85) |
|  | Model 2 | ref | 0.91(0.82,1.00) | 0.83(0.75,0.92) | 0.84(0.76,0.94) |
| DII |  |  |  |  |  |
|  | Model 1 | ref | 0.91(0.85,0.97) | 1.01(0.95,1.07) | 1.28(1.20,1.36) |
|  | Model 2 | ref | 0.94(0.88,1.00) | 0.99(0.93,1.06) | 1.08(1.01,1.14) |

Model 1: Results were adjusted for age and sex.

Model 2: Further adjustment for BMI, birth weight, physical activity, smoking, drinking, townsend deprivation index, employment, family history of diabetes, cholesterol-lowering drug use and hypertension drug use on the basis of model 1.

AHEI:Alternative Healthy Eating Index; DII:Dietary Inflammatory Index; T2D:Type 2 diabetes; BMI:body mass index.

S-Table2. Analysis of the additive interaction between maternal smoking and nutrition factors and T2D.

| Category of exposure | | RERI (95% CI) | AP (95% CI) |
| --- | --- | --- | --- |
| DII | Maternal smoking |  |  |
|  | No | Ref | Ref |
|  | Yes | 0.09 (-0.05,0.23) | 0.08 (-0.04,0.19) |
| AHEI | Maternal smoking |  |  |
|  | No | Ref | Ref |
|  | Yes | -0.29 (-0.55,-0.03) | -0.28 (-0.56,-0.01) |
| T2D_PRS | Maternal smoking |  |  |
|  | No | Ref | Ref |
|  | Yes | 0.05 (-0.06,0.17) | 0.03 (-0.03,0.08) |
| β-cell_PRS | Maternal smoking |  |  |
|  | No | Ref | Ref |
|  | Yes | 0.18 (0.06,0.30) | 0.08 (0.03,0.14) |
| Proinsulin_PRS | Maternal smoking |  |  |
|  | No | Ref | Ref |
|  | Yes | 0.08 (-0.03,0.18) | 0.05 (-0.02,0.11) |
| Obesity_PRS | Maternal smoking |  |  |
|  | No | Ref | Ref |
|  | Yes | 0.08 (-0.02,0.17) | 0.05 (-0.01,0.12) |
| Lipodystroph_PRS | Maternal smoking |  |  |
|  | No | Ref | Ref |
|  | Yes | 0.16 (0.04,0.28) | 0.08 (0.02,0.14) |
| Liver function_PRS | Maternal smoking |  |  |
|  | No | Ref | Ref |
|  | Yes | -0.03 (-0.14,0.07) | -0.02 (-0.09,0.05) |
| HbA1c_PRS | Maternal smoking |  |  |
|  | No | Ref | Ref |
|  | Yes | 0.13 (0.01,0.25) | 0.06 (0.01,0.12) |
| Breastfeeding | Maternal smoking |  |  |
|  | No | Ref | Ref |
|  | Yes | -0.04 (-0.19,0.10) | -0.04 (-0.19,0.1) |

Total 424 SNPs that passed quality control to derive the PRS for T2D. For each SNP, the individual's score was determined by the number of risk alleles they carried (0, 1, or 2). PRS=(β_1_×SNP_1_+β_2_×SNP_2_ +...+β_102_×SNP_102_) × (424/sum of the β coefficients), where βi represents the effect value of the ith SNP. T2D:Type 2 diabetes; SNPs: single nucleotide polymorphisms; PRS:Polygenic risk score; RERI:the relative excess risk due to interaction; AP:the attributable proportion due to interaction.

S-Table 3. The association between genetic risk scores and T2D

| Characters | Model | Q1 | Q2 | Q3 | Q4 |
| --- | --- | --- | --- | --- | --- |
| HbA1c_PRS |  |  |  |  |  |
|  | Model 1 | ref | 1.27(1.22,1.32) | 1.49(1.43,1.55) | 1.88(1.81,1.95) |
|  | Model 2 | ref | 1.24(1.19,1.29) | 1.44(1.39,1.5) | 1.77(1.71,1.84) |
| β-cell_PRS |  |  |  |  |  |
|  | Model 1 | ref | 1.32(1.27,1.38) | 1.57(1.51,1.64) | 2.06(1.98,2.14) |
|  | Model 2 | ref | 1.29(1.24,1.34) | 1.51(1.45,1.57) | 1.86(1.79,1.94) |
| Proinsulin_PRS |  |  |  |  |  |
|  | Model 1 | ref | 1.13(1.08,1.17) | 1.27(1.22,1.32) | 1.67(1.61,1.73) |
|  | Model 2 | ref | 1.11(1.06,1.15) | 1.20(1.16,1.25) | 1.53(1.47,1.59) |
| Obesity_PRS |  |  |  |  |  |
|  | Model 1 | ref | 1.11(1.07,1.15) | 1.19(1.15,1.24) | 1.35(1.30,1.40) |
|  | Model 2 | ref | 1.08(1.04,1.12) | 1.12(1.08,1.16) | 1.21(1.17,1.26) |
| Lipodystroph_PRS |  |  |  |  |  |
|  | Model 1 | ref | 1.26(1.21,1.31) | 1.49(1.43,1.55) | 1.88(1.81,1.95) |
|  | Model 2 | ref | 1.24(1.19,1.29) | 1.44(1.39,1.50) | 1.80(1.73,1.87) |
| Liver function_PRS |  |  |  |  |  |
|  | Model 1 | ref | 1.19(1.14,1.24) | 1.30(1.25,1.36) | 1.56(1.50,1.62) |
|  | Model 2 | ref | 1.17(1.12,1.21) | 1.24(1.19,1.29) | 1.39(1.34,1.45) |

Model 1: Results were adjusted for age and sex.

Model 2: Further adjustment for BMI, birth weight, physical activity, smoking, drinking, townsend deprivation index, employment, family history of diabetes, cholesterol-lowering drug use and hypertension drug use on the basis of model 1.

BMI, body mass index; PRS:Polygenic risk score

S-Table 4. The differences for the baseline characteristics between participants with and without information of maternal smoking around birth and breastfeeding

| Characteristics | Breastfeeding and maternal smoking | | | P-value |
| --- | --- | --- | --- | --- |
|  | Total | Non-missing | Missing data |  |
|  | N = 472,301 | N = 328,820 | N = 143,481 |  |
| Age, mean (SD) | 56.5 (8.1) | 55.7 (8.1) | 58.4 (7.6) | <0.001 |
| Sex, % |  |  |  | <0.001 |
| men | 258,663 (54.8%) | 190,526 (57.9%) | 68,137 (47.5%) |  |
| women | 213,638 (45.2%) | 138,294 (42.1%) | 75,344 (52.5%) |  |
| Race (%) |  |  |  | <0.001 |
| Non-white ethnicity | 22,263 (4.7%) | 18,398 (5.6%) | 3,865 (2.7%) |  |
| White ethnicity | 450,038 (95.3%) | 310,422 (94.4%) | 139,616 (97.3%) |  |
| Smoking status, % |  |  |  | <0.001 |
| No | 259,241 (54.9%) | 185,966 (56.6%) | 73,275 (51.1%) |  |
| Previous | 164,497 (34.8%) | 110,519 (33.6%) | 53,978 (37.6%) |  |
| Current | 48,563 (10.3%) | 32,335 (9.8%) | 16,228 (11.3%) |  |
| Drinking status, % |  |  |  | <0.001 |
| No | 19,425 (4.1%) | 14,400 (4.4%) | 5,025 (3.5%) |  |
| Previous | 16,416 (3.5%) | 11,118 (3.4%) | 5,298 (3.7%) |  |
| Current | 436,460 (92.4%) | 303,302 (92.2%) | 133,158 (92.8%) |  |
| Employment (%) |  |  |  | <0.001 |
| In paid employment or self-employed | 274,331 (58.1%) | 200,734 (61.0%) | 73,597 (51.3%) |  |
| Retired | 157,532 (33.4%) | 99,189 (30.2%) | 58,343 (40.7%) |  |
| Others | 40,438 (8.6%) | 28,897 (8.8%) | 11,541 (8.0%) |  |
| MET-min/week ≥600 | 299,773 (63.5%) | 214,152 (65.1%) | 85,621 (59.7%) | <0.001 |
| BMI, kg/m2 |  |  |  | <0.001 |
| ＜25 | 157,700 (33.4%) | 113,351 (34.5%) | 44,349 (30.9%) |  |
| 25-29.9 | 200,675 (42.5%) | 137,447 (41.8%) | 63,228 (44.1%) |  |
| ≥30 | 113,926 (24.1%) | 78,022 (23.7%) | 35,904 (25.0%) |  |
| Townsend deprivation index, mean (SD) | -1.38 (3.04) | -1.40 (3.03) | -1.34 (3.06) | <0.001 |
| Prevalent Hypertension, % | 26,128 (5.5%) | 18,172 (5.5%) | 7,956 (5.5%) | <0.001 |
| Cholesterol-lowering drug use (%) | 32,208 (6.8%) | 21,783 (6.6%) | 10,425 (7.3%) | <0.001 |
| Family history of diabetes (%) | 88,078 (18.6%) | 63,471 (19.3%) | 24,607 (17.2%) | <0.001 |

Continuous variables are presented as Mean±SD. Categorical variables are presented as numbers (%, percentage). BMI, body mass index; SD, standard deviation

S-Table 5. The differences for the baseline characteristics between participants with and without information of maternal smoking around birth and breastfeeding after PSM matching

| Characteristics | Breastfeeding and maternal smoking | | P-value |
| --- | --- | --- | --- |
|  | Non-missing | missing data |  |
|  | N = 136,479 | N = 134,775 |  |
| Age, years | 58.0(8.0) | 58.0(8.0) | 0.900 |
| Sex, % |  |  | 0.017 |
| men | 66,391 (48.6%) | 64,942 (48.2%) |  |
| women | 70,088 (51.4%) | 69,833 (51.8%) |  |
| Race (%) |  |  | 0.051 |
| Non-white ethnicity | 3,667 (2.7%) | 3,460 (2.6%) |  |
| White ethnicity | 132,812 (97.3%) | 131,315 (97.4%) |  |
| Smoking status, % |  |  | 0.800 |
| No | 70,803 (51.9%) | 69,765 (51.8%) |  |
| Previous | 50,585 (37.1%) | 50,039 (37.1%) |  |
| Current | 15,091 (11.1%) | 14,971 (11.1%) |  |
| Drinking status, % |  |  | 0.800 |
| No | 4,612 (3.4%) | 4,605 (3.4%) |  |
| Previous | 4,763 (3.5%) | 4,720 (3.5%) |  |
| Current | 127,104 (93.1%) | 125,450 (93.1%) |  |
| Employment (%) |  |  | 0.800 |
| In paid employment or self-employed | 71,029 (52.0%) | 70,256 (52.1%) |  |
| Retired | 54,861 (40.2%) | 54,027 (40.1%) |  |
| Others | 10,589 (7.8%) | 10,492 (7.8%) |  |
| Physical activity (%), MET-min/week ≥600 |  |  | 0.500 |
| BMI, kg/m^2^ |  |  | 0.700 |
| ＜25 | 43,050 (31.5%) | 42,715 (31.7%) |  |
| 25-29.9 | 60,648 (44.4%) | 59,824 (44.4%) |  |
| ≥30 | 32,781 (24.0%) | 32,236 (23.9%) |  |
| Townsend deprivation index, mean (SD) | -1.39(3.04) | -1.39(3.03) | 0.300 |
| Prevalent Hypertension, % | 7,890 (5.8%) | 7,608 (5.6%) | 0.037 |
| Cholesterol-lowering drug use (%) | 9,430 (6.9%) | 9,084 (6.7%) | 0.031 |
| Family history of diabetes (%) | 23,244 (17.0%) | 23,055 (17.1%) | 0.046 |

Continuous variables are presented as Mean(SD). Categorical variables are presented as numbers (%, percentage). BMI, body mass index.
