# Supplementary figures and images for: Maternal smoking, nutritional factors at different life stage, and the risk of incident type 2 diabetes: a prospective study of the UK Biobank
Source: BMC Med. 2024 Feb 2;22:50. doi: 10.1186/s12916-024-03256-8 (PMC10835913; doi:10.1186/s12916-024-03256-8)

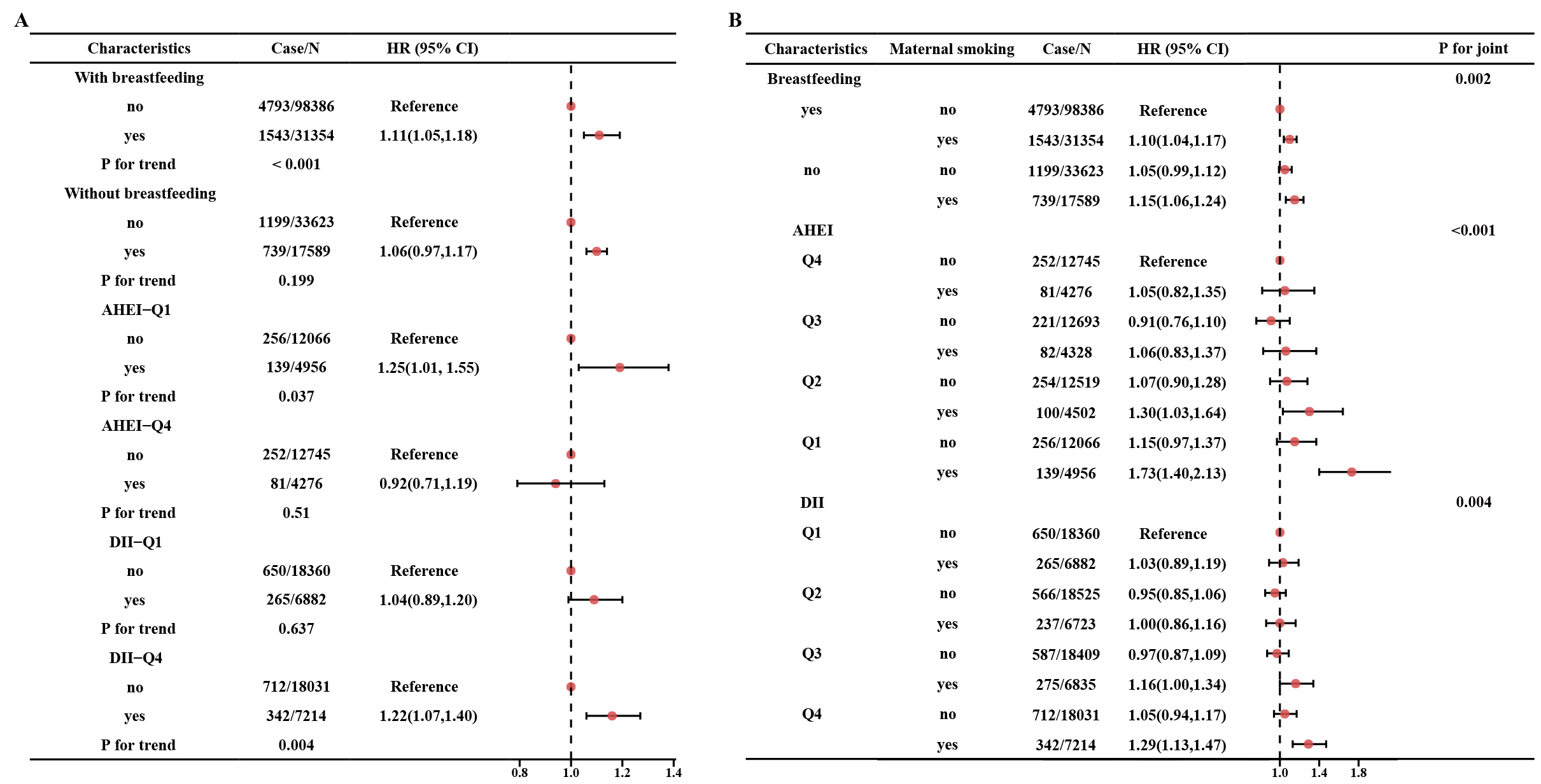

Supplement: Supplementary file 2 — Additional file 2: Fig. S1. The association between MSAB and T2D stratified by various levels of breastfeeding, AHEI and DII, excluding individuals with follow-up periods less than two years. Fig. S2. The association between MSAB and T2D stratified by different genetic risk scores, excluding individuals with follow-up periods less than two years. Fig. S3. Joint analysis of genetic risk scores and MSAB in relation to T2D, excluding individuals with follow-up periods less than two years. Fig. S4. The association between MSAB and T2D stratified by various levels of breastfeeding, AHEI and DII in a non-smoking population. Fig. S5. The association between MSAB and T2D stratified by different genetic risk scores in a non-smoking population. Fig. S6. Joint analysis of genetic risk scores and MSAB in relation to T2D in a non-smoking population. Fig. S7. The association between MSAB and T2D stratified by various levels of breastfeeding, AHEI and DII in the selected population after propensity score matching. Fig. S8. The association between MSAB and T2D stratified by different genetic risk scores in the selected population after propensity score matching. Fig. S9. Joint analysis of genetic risk scores and MSAB in relation to T2D in the selected population after propensity score matching. [file 12916_2024_3256_MOESM2_ESM.zip › S-Figure 1R2.jpg]

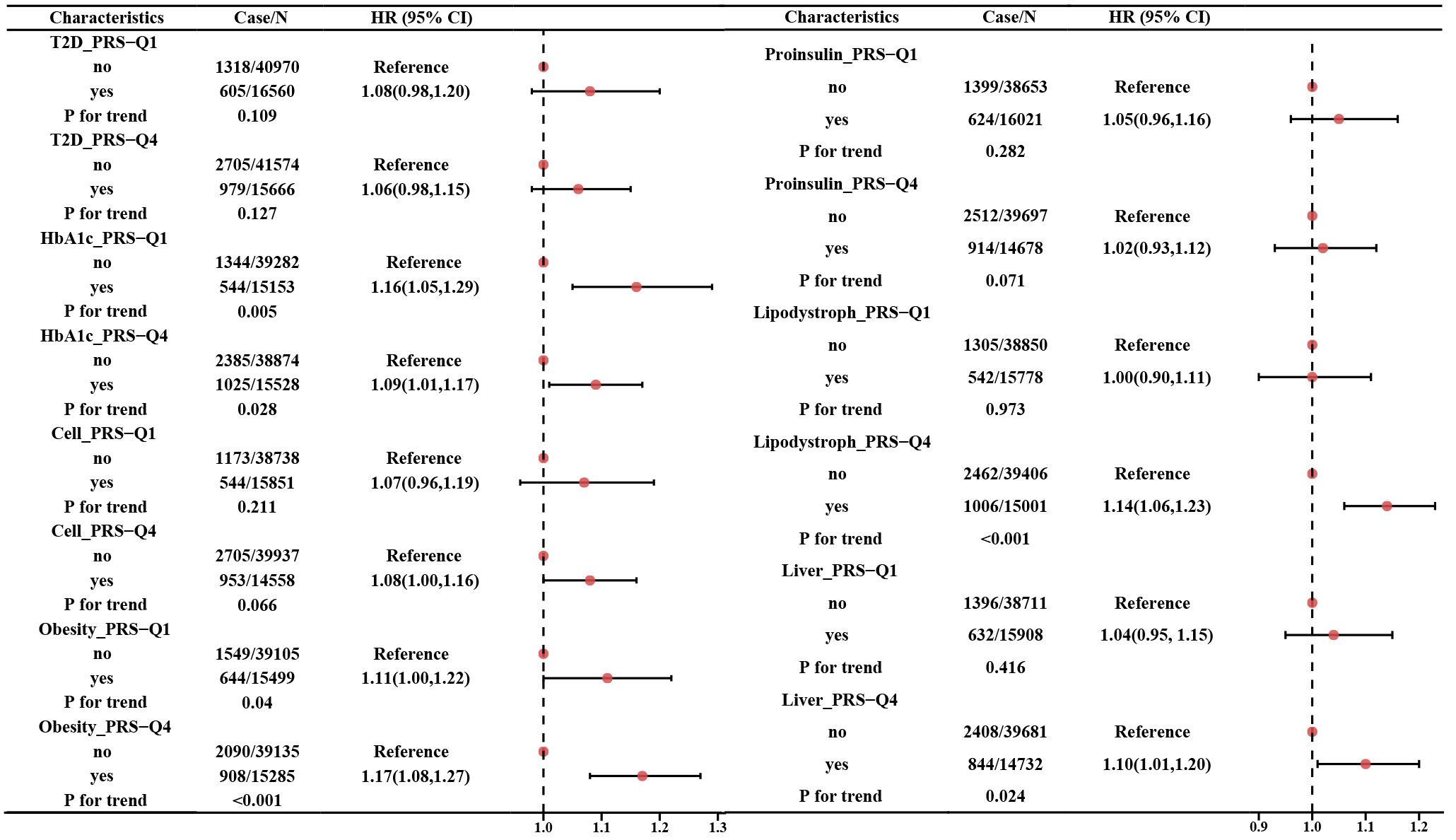

Supplement: Supplementary file 2 — Additional file 2: Fig. S1. The association between MSAB and T2D stratified by various levels of breastfeeding, AHEI and DII, excluding individuals with follow-up periods less than two years. Fig. S2. The association between MSAB and T2D stratified by different genetic risk scores, excluding individuals with follow-up periods less than two years. Fig. S3. Joint analysis of genetic risk scores and MSAB in relation to T2D, excluding individuals with follow-up periods less than two years. Fig. S4. The association between MSAB and T2D stratified by various levels of breastfeeding, AHEI and DII in a non-smoking population. Fig. S5. The association between MSAB and T2D stratified by different genetic risk scores in a non-smoking population. Fig. S6. Joint analysis of genetic risk scores and MSAB in relation to T2D in a non-smoking population. Fig. S7. The association between MSAB and T2D stratified by various levels of breastfeeding, AHEI and DII in the selected population after propensity score matching. Fig. S8. The association between MSAB and T2D stratified by different genetic risk scores in the selected population after propensity score matching. Fig. S9. Joint analysis of genetic risk scores and MSAB in relation to T2D in the selected population after propensity score matching. [file 12916_2024_3256_MOESM2_ESM.zip › S-Figure 2R2.jpg]

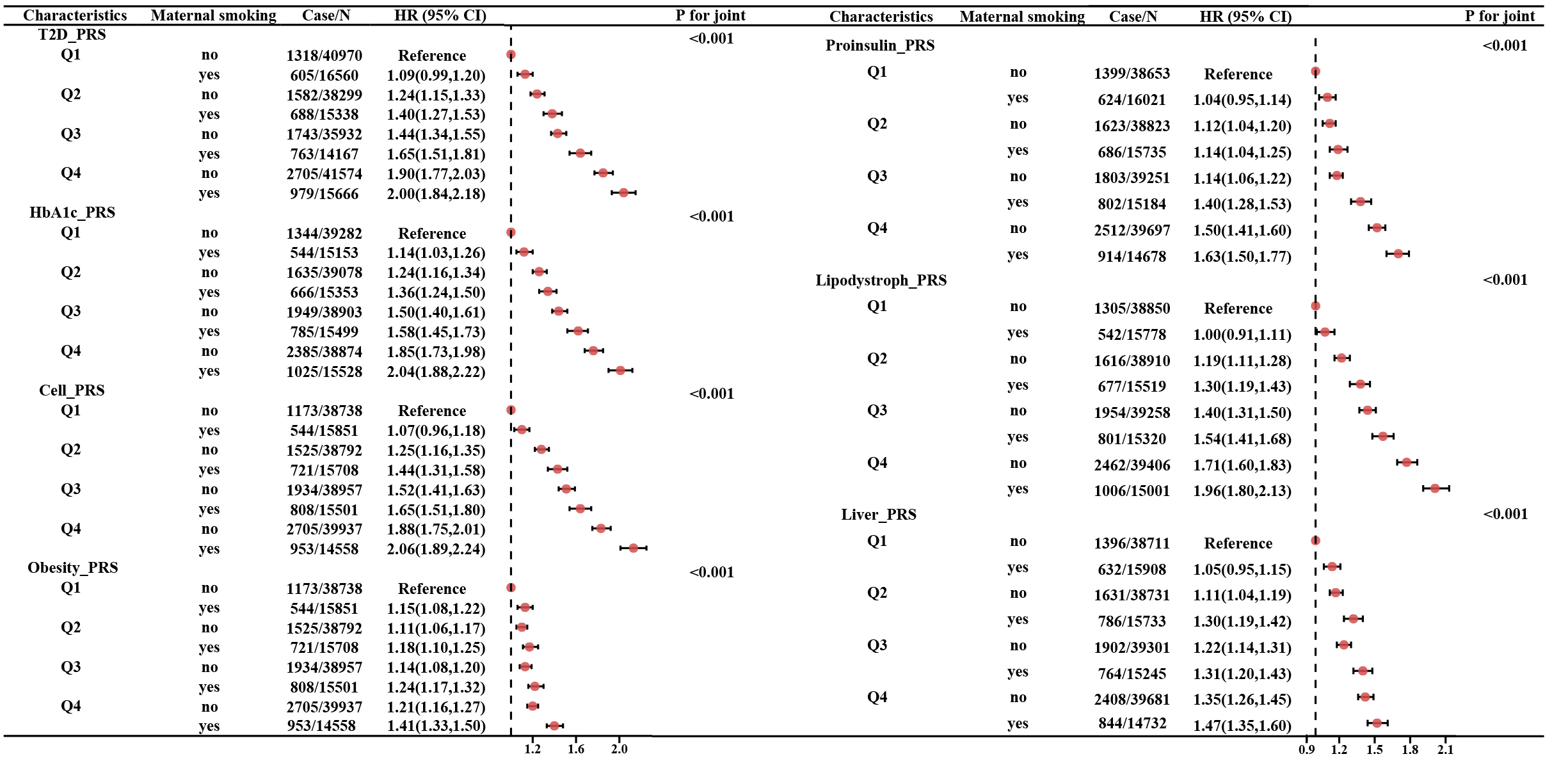

Supplement: Supplementary file 2 — Additional file 2: Fig. S1. The association between MSAB and T2D stratified by various levels of breastfeeding, AHEI and DII, excluding individuals with follow-up periods less than two years. Fig. S2. The association between MSAB and T2D stratified by different genetic risk scores, excluding individuals with follow-up periods less than two years. Fig. S3. Joint analysis of genetic risk scores and MSAB in relation to T2D, excluding individuals with follow-up periods less than two years. Fig. S4. The association between MSAB and T2D stratified by various levels of breastfeeding, AHEI and DII in a non-smoking population. Fig. S5. The association between MSAB and T2D stratified by different genetic risk scores in a non-smoking population. Fig. S6. Joint analysis of genetic risk scores and MSAB in relation to T2D in a non-smoking population. Fig. S7. The association between MSAB and T2D stratified by various levels of breastfeeding, AHEI and DII in the selected population after propensity score matching. Fig. S8. The association between MSAB and T2D stratified by different genetic risk scores in the selected population after propensity score matching. Fig. S9. Joint analysis of genetic risk scores and MSAB in relation to T2D in the selected population after propensity score matching. [file 12916_2024_3256_MOESM2_ESM.zip › S-Figure 3R2.jpg]

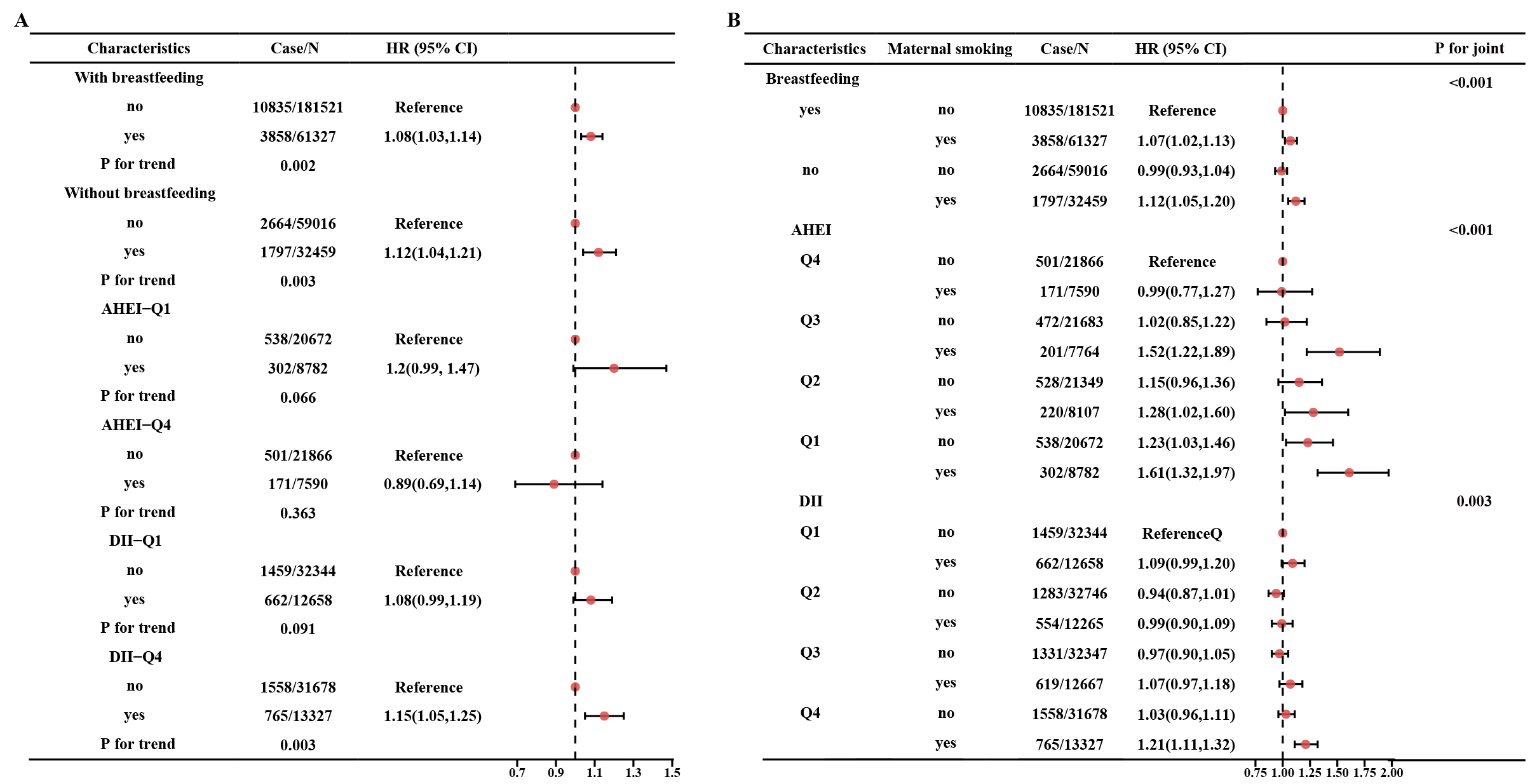

Supplement: Supplementary file 2 — Additional file 2: Fig. S1. The association between MSAB and T2D stratified by various levels of breastfeeding, AHEI and DII, excluding individuals with follow-up periods less than two years. Fig. S2. The association between MSAB and T2D stratified by different genetic risk scores, excluding individuals with follow-up periods less than two years. Fig. S3. Joint analysis of genetic risk scores and MSAB in relation to T2D, excluding individuals with follow-up periods less than two years. Fig. S4. The association between MSAB and T2D stratified by various levels of breastfeeding, AHEI and DII in a non-smoking population. Fig. S5. The association between MSAB and T2D stratified by different genetic risk scores in a non-smoking population. Fig. S6. Joint analysis of genetic risk scores and MSAB in relation to T2D in a non-smoking population. Fig. S7. The association between MSAB and T2D stratified by various levels of breastfeeding, AHEI and DII in the selected population after propensity score matching. Fig. S8. The association between MSAB and T2D stratified by different genetic risk scores in the selected population after propensity score matching. Fig. S9. Joint analysis of genetic risk scores and MSAB in relation to T2D in the selected population after propensity score matching. [file 12916_2024_3256_MOESM2_ESM.zip › S-Figure 4R2.jpg]

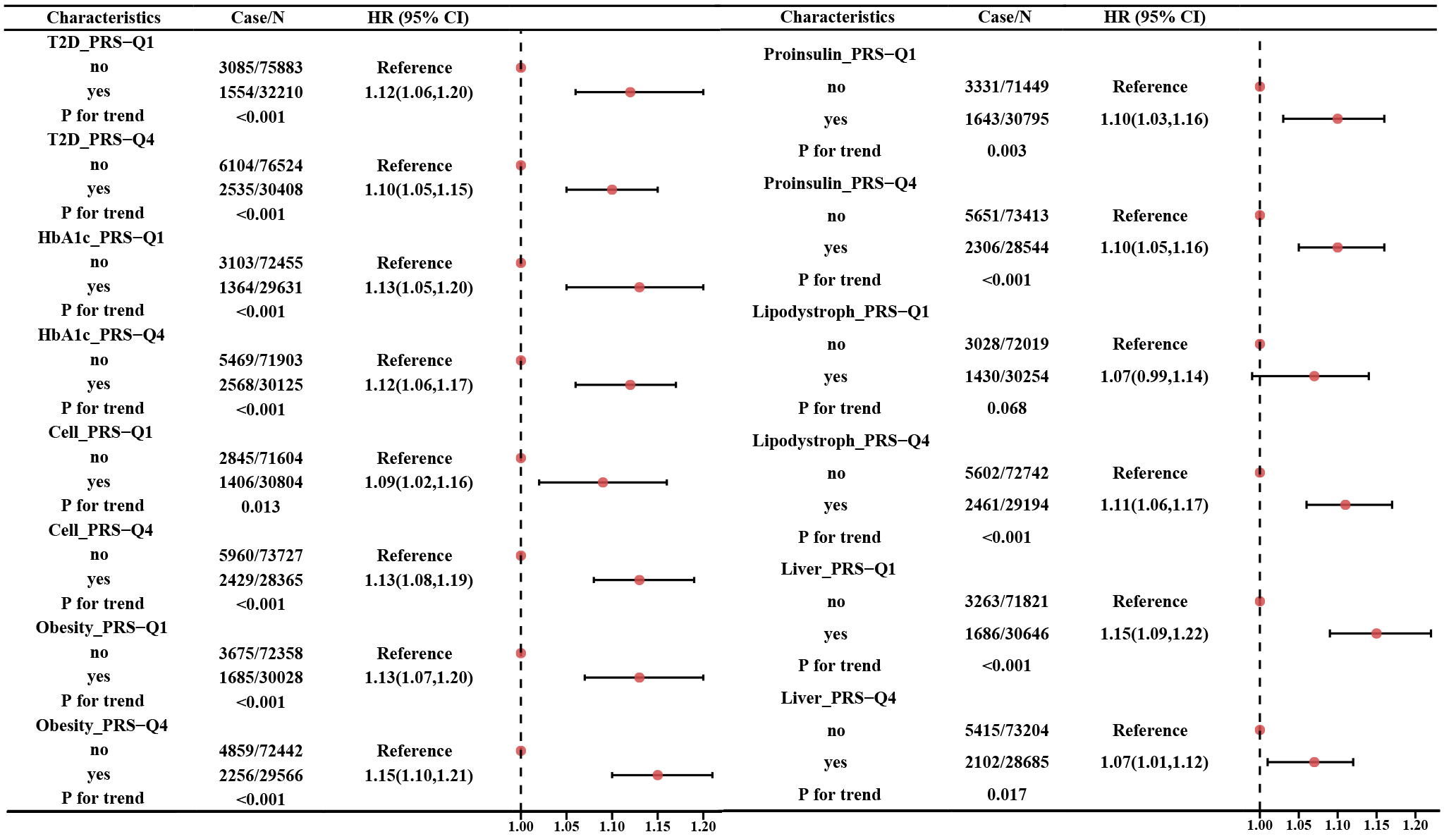

Supplement: Supplementary file 2 — Additional file 2: Fig. S1. The association between MSAB and T2D stratified by various levels of breastfeeding, AHEI and DII, excluding individuals with follow-up periods less than two years. Fig. S2. The association between MSAB and T2D stratified by different genetic risk scores, excluding individuals with follow-up periods less than two years. Fig. S3. Joint analysis of genetic risk scores and MSAB in relation to T2D, excluding individuals with follow-up periods less than two years. Fig. S4. The association between MSAB and T2D stratified by various levels of breastfeeding, AHEI and DII in a non-smoking population. Fig. S5. The association between MSAB and T2D stratified by different genetic risk scores in a non-smoking population. Fig. S6. Joint analysis of genetic risk scores and MSAB in relation to T2D in a non-smoking population. Fig. S7. The association between MSAB and T2D stratified by various levels of breastfeeding, AHEI and DII in the selected population after propensity score matching. Fig. S8. The association between MSAB and T2D stratified by different genetic risk scores in the selected population after propensity score matching. Fig. S9. Joint analysis of genetic risk scores and MSAB in relation to T2D in the selected population after propensity score matching. [file 12916_2024_3256_MOESM2_ESM.zip › S-Figure 5R2.jpg]

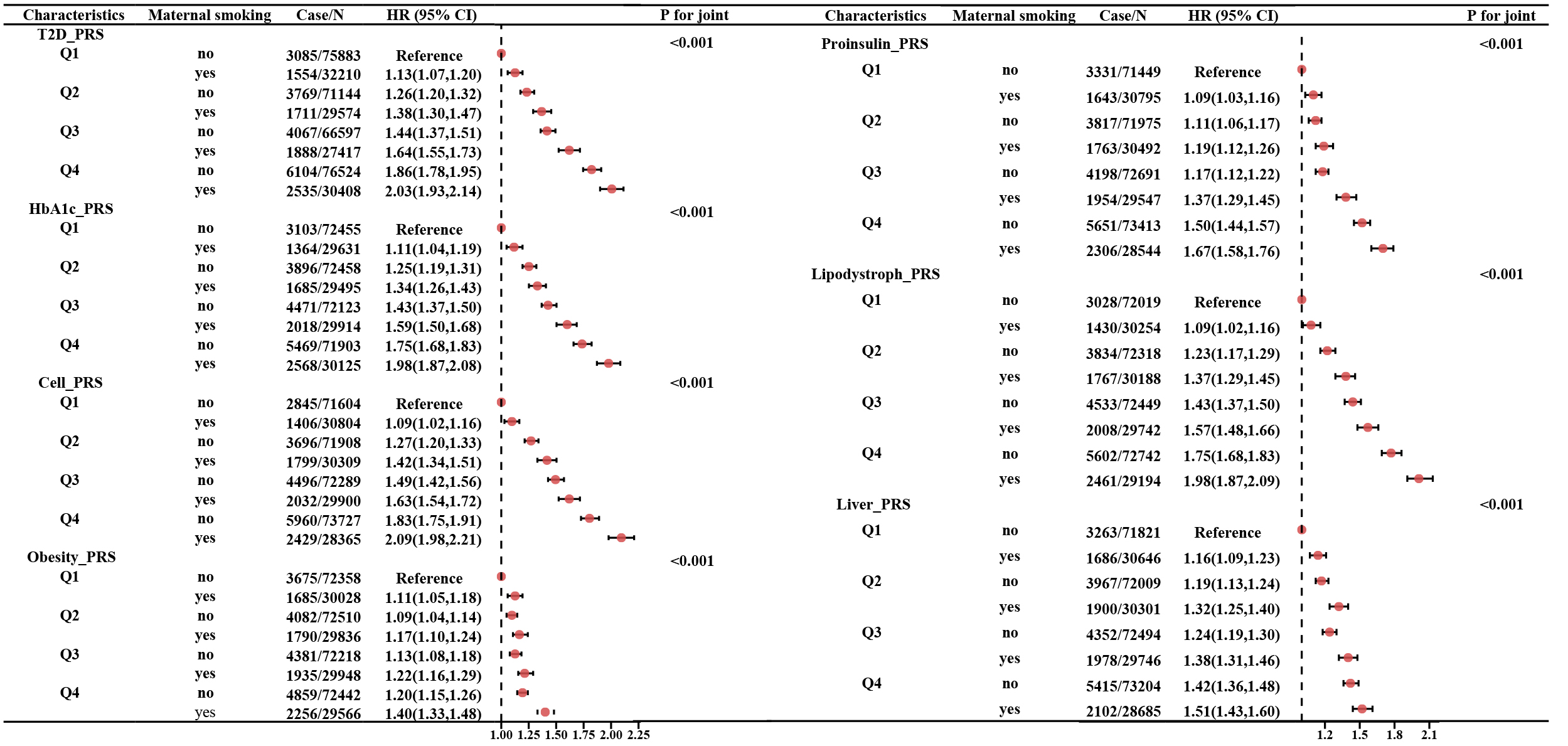

Supplement: Supplementary file 2 — Additional file 2: Fig. S1. The association between MSAB and T2D stratified by various levels of breastfeeding, AHEI and DII, excluding individuals with follow-up periods less than two years. Fig. S2. The association between MSAB and T2D stratified by different genetic risk scores, excluding individuals with follow-up periods less than two years. Fig. S3. Joint analysis of genetic risk scores and MSAB in relation to T2D, excluding individuals with follow-up periods less than two years. Fig. S4. The association between MSAB and T2D stratified by various levels of breastfeeding, AHEI and DII in a non-smoking population. Fig. S5. The association between MSAB and T2D stratified by different genetic risk scores in a non-smoking population. Fig. S6. Joint analysis of genetic risk scores and MSAB in relation to T2D in a non-smoking population. Fig. S7. The association between MSAB and T2D stratified by various levels of breastfeeding, AHEI and DII in the selected population after propensity score matching. Fig. S8. The association between MSAB and T2D stratified by different genetic risk scores in the selected population after propensity score matching. Fig. S9. Joint analysis of genetic risk scores and MSAB in relation to T2D in the selected population after propensity score matching. [file 12916_2024_3256_MOESM2_ESM.zip › S-Figure 6R2.jpg]

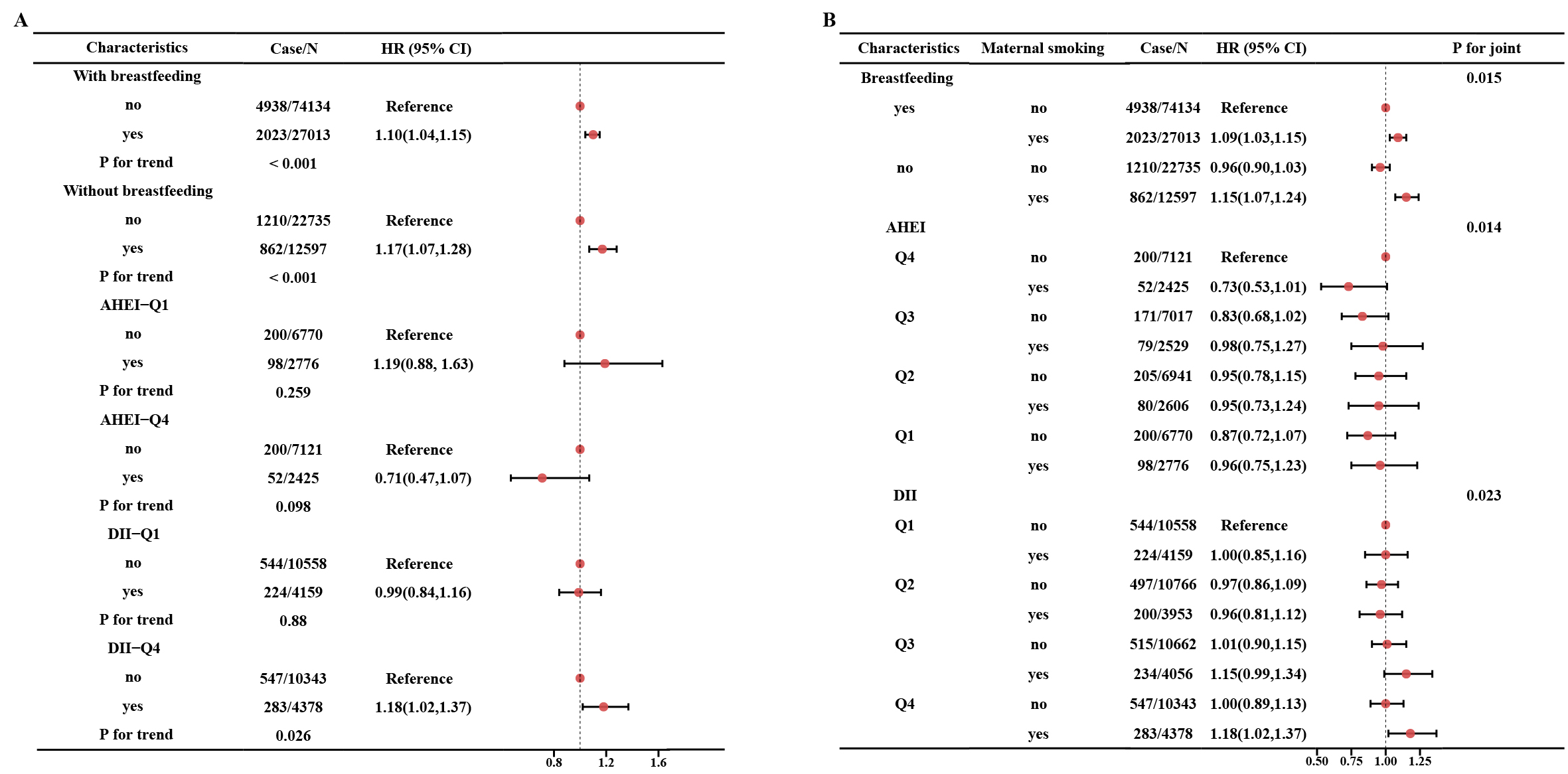

Supplement: Supplementary file 2 — Additional file 2: Fig. S1. The association between MSAB and T2D stratified by various levels of breastfeeding, AHEI and DII, excluding individuals with follow-up periods less than two years. Fig. S2. The association between MSAB and T2D stratified by different genetic risk scores, excluding individuals with follow-up periods less than two years. Fig. S3. Joint analysis of genetic risk scores and MSAB in relation to T2D, excluding individuals with follow-up periods less than two years. Fig. S4. The association between MSAB and T2D stratified by various levels of breastfeeding, AHEI and DII in a non-smoking population. Fig. S5. The association between MSAB and T2D stratified by different genetic risk scores in a non-smoking population. Fig. S6. Joint analysis of genetic risk scores and MSAB in relation to T2D in a non-smoking population. Fig. S7. The association between MSAB and T2D stratified by various levels of breastfeeding, AHEI and DII in the selected population after propensity score matching. Fig. S8. The association between MSAB and T2D stratified by different genetic risk scores in the selected population after propensity score matching. Fig. S9. Joint analysis of genetic risk scores and MSAB in relation to T2D in the selected population after propensity score matching. [file 12916_2024_3256_MOESM2_ESM.zip › S-Figure 7R2.jpg]

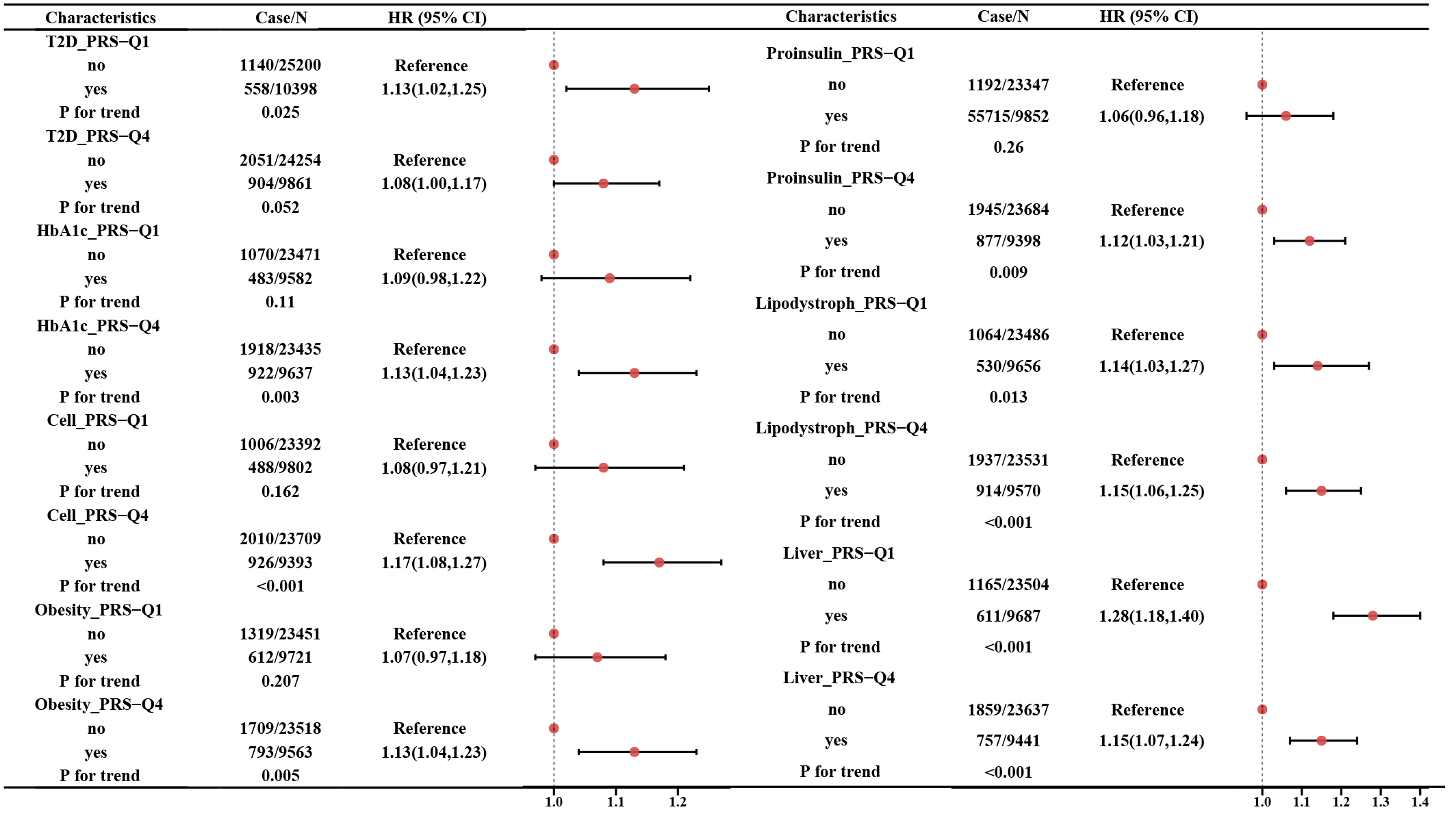

Supplement: Supplementary file 2 — Additional file 2: Fig. S1. The association between MSAB and T2D stratified by various levels of breastfeeding, AHEI and DII, excluding individuals with follow-up periods less than two years. Fig. S2. The association between MSAB and T2D stratified by different genetic risk scores, excluding individuals with follow-up periods less than two years. Fig. S3. Joint analysis of genetic risk scores and MSAB in relation to T2D, excluding individuals with follow-up periods less than two years. Fig. S4. The association between MSAB and T2D stratified by various levels of breastfeeding, AHEI and DII in a non-smoking population. Fig. S5. The association between MSAB and T2D stratified by different genetic risk scores in a non-smoking population. Fig. S6. Joint analysis of genetic risk scores and MSAB in relation to T2D in a non-smoking population. Fig. S7. The association between MSAB and T2D stratified by various levels of breastfeeding, AHEI and DII in the selected population after propensity score matching. Fig. S8. The association between MSAB and T2D stratified by different genetic risk scores in the selected population after propensity score matching. Fig. S9. Joint analysis of genetic risk scores and MSAB in relation to T2D in the selected population after propensity score matching. [file 12916_2024_3256_MOESM2_ESM.zip › S-Figure 8R2.jpg]

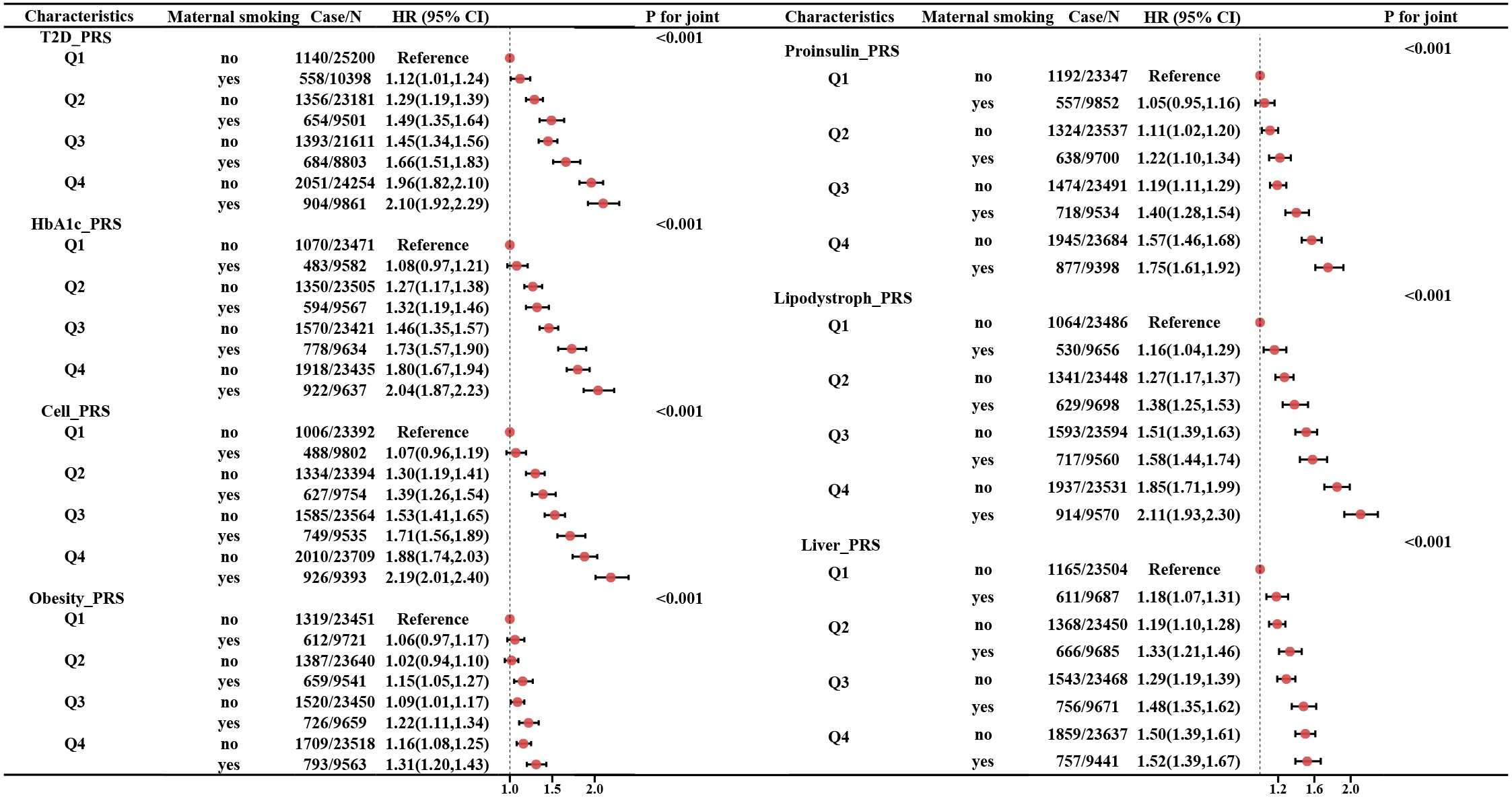

Supplement: Supplementary file 2 — Additional file 2: Fig. S1. The association between MSAB and T2D stratified by various levels of breastfeeding, AHEI and DII, excluding individuals with follow-up periods less than two years. Fig. S2. The association between MSAB and T2D stratified by different genetic risk scores, excluding individuals with follow-up periods less than two years. Fig. S3. Joint analysis of genetic risk scores and MSAB in relation to T2D, excluding individuals with follow-up periods less than two years. Fig. S4. The association between MSAB and T2D stratified by various levels of breastfeeding, AHEI and DII in a non-smoking population. Fig. S5. The association between MSAB and T2D stratified by different genetic risk scores in a non-smoking population. Fig. S6. Joint analysis of genetic risk scores and MSAB in relation to T2D in a non-smoking population. Fig. S7. The association between MSAB and T2D stratified by various levels of breastfeeding, AHEI and DII in the selected population after propensity score matching. Fig. S8. The association between MSAB and T2D stratified by different genetic risk scores in the selected population after propensity score matching. Fig. S9. Joint analysis of genetic risk scores and MSAB in relation to T2D in the selected population after propensity score matching. [file 12916_2024_3256_MOESM2_ESM.zip › S-Figure 9R2.jpg]
